# Splenogonadal fusion: a case report and review of the literature

**DOI:** 10.1186/s12894-021-00781-z

**Published:** 2021-02-03

**Authors:** Guangjie Chen, Xiaohao Wang, Yijun Zhao, Linfeng Zhu, Daxing Tang

**Affiliations:** Department of Urology, The Children’s Hospital, Zhejiang University, School of Medicine, National Clinical Research Center for Child Health, 57 Zhugan Xiang, Hangzhou, 310053 China

**Keywords:** Splenogondal fusion (SGF), Orchiopexy, Pediatric, Case report

## Abstract

**Background:**

Splenogondal fusion (SGF) is a rare congenital anomaly characterized by abnormal association between the splenic tissue and the gonads or mesonephric remnants. SGF that requires separate two-stage laparoscopic staged Fowler-Stephen orchiopexy on both the left and right sides is extremely rare. SGF could be misdiagnosed as testicular malignancy and leads to unnecessary orchiectomy.

**Case presentation:**

This is a case of an 8-month old male infant presented with bilateral cryptorchidism, B-mode ultrasound visualized the left and right testes in the lower abdominal cavity and the upper margin of the left testicle as a hypoechoic mass extending to the spleen, indicating an undescended right testis and possible SGF on the left side. Single-site laparoscopic examination confirmed the diagnosis of SGF on the left side and an undescended right testis. As both testes were high and the right spermatic vessel was poorly developed and short, a routine single stage orchiopexy would be difficult and risky, therefore, separate two-stage laparoscopic staged Fowler-Stephen orchiopexies for both sides were implemented. Stage 1 of the staged Fowler-Stephen orchiopexy for the right side was performed first without treating the left side, Stage 2 for the right side, separation of the left testis from the spleen as well as Stage 1 for the left side were performed 7 months later, and Stage 2 for the left side was performed 7 months after that. Follow-up ultrasound 1 year after the surgery revealed no obvious abnormalities in the shapes of the testes or their blood supply. This treatment strategy prevented unnecessary orchiectomy.

**Conclusions:**

We reported a rare case of SGF that needed separate two-stage laparoscopic staged Fowler-Stephen orchiopexies for both sides, and a review of the recent literature. SGF is a rare congenital anomaly often diagnosed incidentally during exploration/surgery for scrotal swelling/mass, cryptorchidism or inguinal hernia in young patients. Surgeons, especially pediatric surgeons should be aware of this rare condition to avoid unnecessary, life-altering radical orchiectomy. When routine single stage orchiopexy is not feasible or risky for either side, separate two-stage laparoscopic staged Fowler-Stephen orchiopexies could be performed on both the left and right sides to avoid unnecessary orchiectomy.

## Background

Splenogonadal fusion (SGF) is a rare benign congenital malformation characterized by an abnormal association between the splenic tissue and the gonads or mesonephric remnants [1–3]. It was first reported by Bostroem in 1883 and the first detailed review of 30 SGF cases was published by Putschar and Manion in 1956 wherein a SGF classification system was established [1–4]. In 1990, Carragher did a comprehensive review of 123 reported SGF cases, of which about 70% were pediatric cases [3]. Subsequently, Malik et al*.* in 2013 published a review of 61 additional SGF cases [5]. Since then, 40 new SGF cases have been reported in English [2, 6–41]. SGF is a condition that usually presents as inguinal hernia, cryptorchidism, or scrotal swelling/mass, and it could be misdiagnosed as testicular malignancy and leads to unnecessary, life-altering orchiectomy [2, 7, 12, 13]. Lack of awareness of this condition is a major reason for its misdiagnosis [2, 7, 12, 13]. SGF cases that present as cryptorchidism are often treated with single stage laparoscopic Fowler-Stephen orchiopexy [11, 30, 32]. In the current study, we report 1 unique SGF case that presented as bilateral cryptorchidism that required separate two-stage laparoscopic staged Fowler-Stephen orchiopexies on both the left and right sides, because performing routine single stage laparoscopic Fowler-Stephen orchiopexy was very difficult and risk. This strategy prevented unnecessary orchiectomy in this patient. A literature review of the 40 newly reported SGF cases [2, 6–41] was also provided. This study was approved by the ethics committee of the Children’s Hospital, Zhejiang University, School of Medicine, National Clinical Research Center for Child Health, and written informed consent was obtained from the patient’s caregiver for the study and the publication of this manuscript and any accompanying images. We present the following case in accordance with the CARE Guideline [42].

## Case presentation

**Fig. 1:**
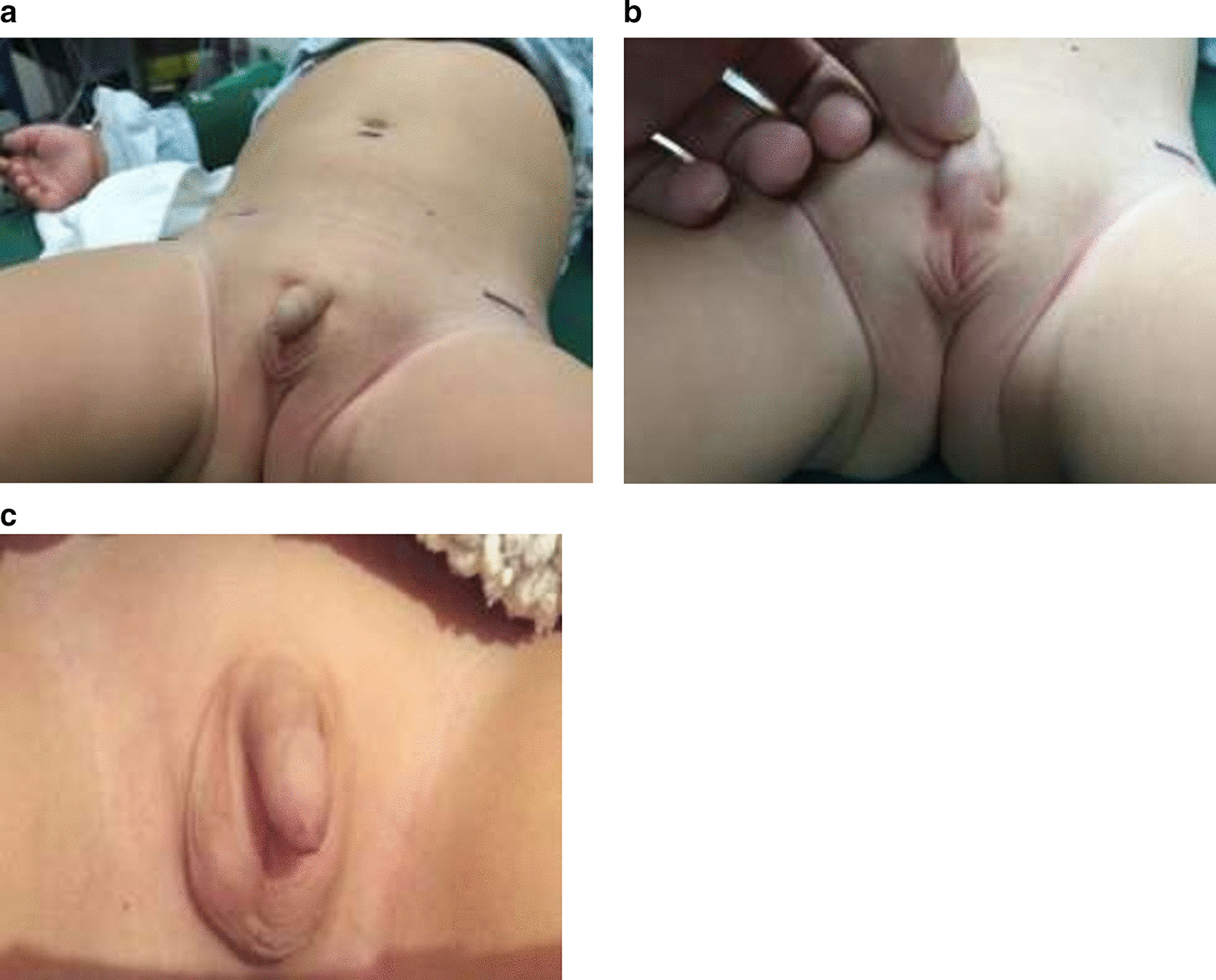
The appearance of an 8-month old male infant’s external genitalia. **a**, **b** Before the surgery; **c** after the surgery

**Fig. 2:**
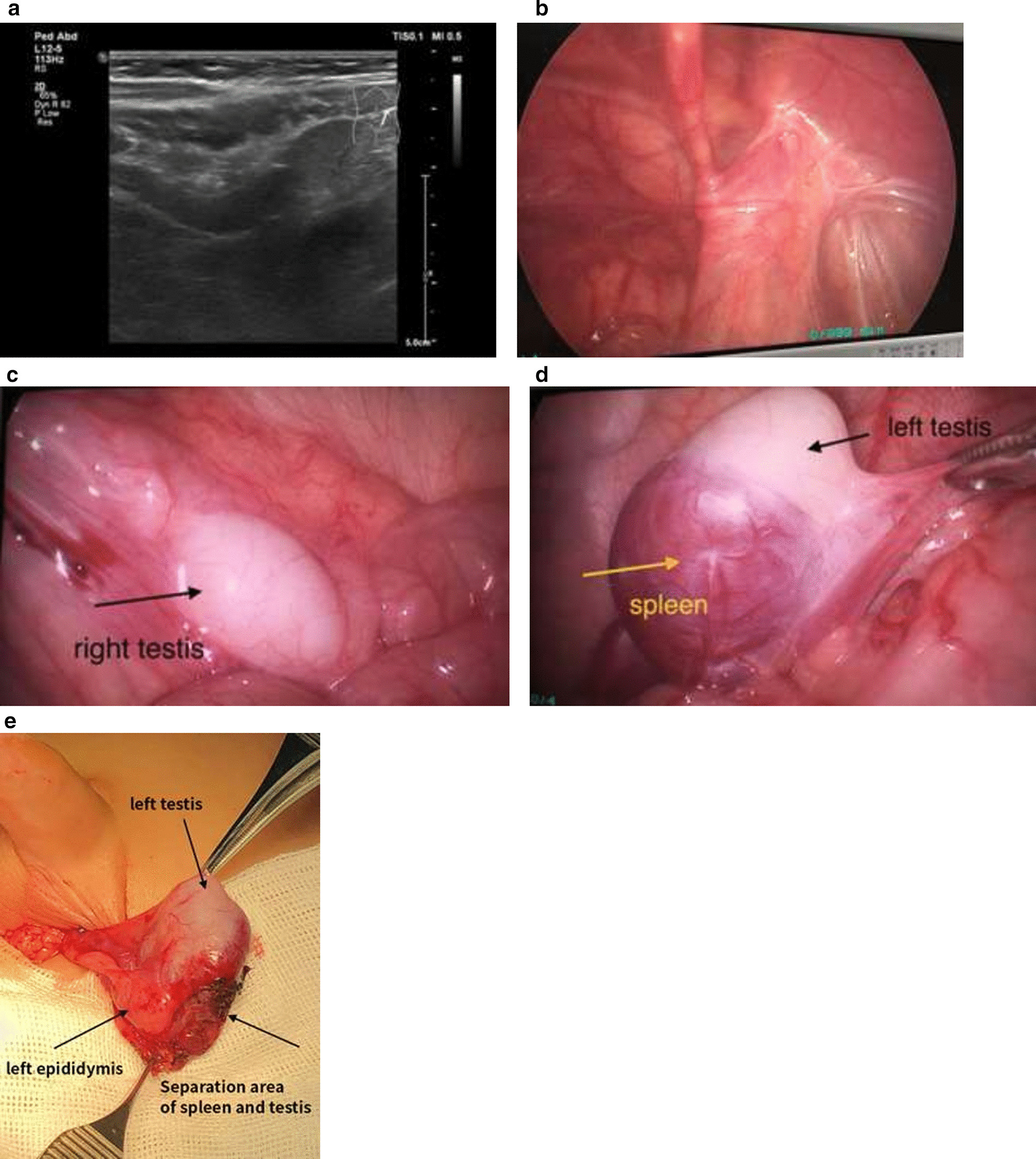
Continuous splenogonadal fusion on the left side and undescended right testis in an 8-month old male infant. **a** Pre-operative B-mode ultrasound revealed possible fusion of the left testis and the kidney; **b** single site laparoscopic examination revealed closed right internal ring and the right testis in the iliac fossa, at about the same height as the junction between the iliac vessel and the right vas deferens; **c** single-site laparoscopic examination visualized the right testis located in the right iliac fossa, approximately 2 cm above the right internal ring, with poorly developed right spermatic vessel; **d** complete fusion of the spleen and the left testis in the left iliac fossa visualized by the single-site laparoscopic examination; **e** the left testis descended into the scrotum after being separated from the spleen

An 8-month old male infant presented with bilateral cryptorchidism since birth was referred to our hospital and hospitalized. His mother had tocolytic treatment prior to his birth. The patient’s medical and family history revealed nothing unusual. The patient had received no prior treatment for the bilateral cryptorchidism. Physical examination showed that the patient was 68.5 cm high, weighed 9.5 kg, had a head circumference of 42.4 cm and was in good general health except for the bilateral cryptorchidism. Examination of the patient’s four limbs and chest, palpation of the spine and musculoskeletal examination revealed no abnormality, and the patient had normal vision and hearing. An X-ray revealed no obvious pulmonary abnormality. The patient had bilateral flat and empty scrotum with no split and there was no obvious inguinal mass on either side (Fig. 1a, b). The patient had a properly developed corpus cavernosum and normal urinary meatus. B-mode ultrasound indicated bilateral cryptorchidism and visualized a left testis of 1.4 × 0.8 cm and a right testis of 1.2 × 0.6 cm in the lower abdominal cavity. In addition, the ultrasound also visualized the upper margin of the left testicle as a hypoechoic mass extending to the spleen. The ultrasound findings suggested an undescended right testis and a possible SGF on the left side (Fig. 2a). The ultrasound did not reveal any abnormality in the patient’s heart, liver or spleen. Magnetic resonance imaging (MRI) visualized no obvious testis on either side and no obvious abnormality in the patient’s heart, spleen or liver. In addition, MRI showed no obvious uterus, ovary or fallopian tube-like structures. As to the patient’s kidneys, both the ultrasound and MRI visualized kidneys with normal sizes and shapes at normal positions, intact renal capsule, and renal pelvis and ureter that were not dilated, In addition, per our routine practice, we assessed the patient’s External Masculinization Score (EMS) to summarize clinical features representing his genitalia and to determine the degree of masculinization of this male infant, he had an EMS score of 10 out of 12 [43]. Overall, our physical examination of the patient ruled out other obvious congenital disorders.

We also screened for genetic variant(s) related to disorder of sex development with panel based next generation sequencing (NGS) test using multiple ligation-dependent probe amplification (MLPA). The NGS test did not reveal any known SGF related genetic variant, although the test did indicate that the patient carries a missense mutation in the gene encoding SET binding protein 1 (SETBP1) (c.2608G>A [p.Gly870Ser]), a causative variation for Schinzel-Giedion syndrome (OMIM #269150) [44], a frameshift or nonsense mutation in SETBP1 known to cause autosomal dominant mental retardation-29 (OMIM #616078) [45], and a mutation in the oligophrenin-1 gene (OPHN1) known to cause X-linked mental retardation with cerebellar hypoplasia (OMIM #300486) [46].

The first laparoscopic surgery was performed on January 11, 2018, single-site laparoscopy revealed closed internal rings on both sides, right testis in the in the iliac fossa, positioned approximately 2 cm above the right internal ring, at about the same height as the junction between the iliac vessel and the ureter (Fig. 2b, c). It also showed that the left testis was about 3.5 cm above the left internal ring and 1.5 cm above the junction between the iliac vessel and the ureter, and that the left testis was fused to the spleen with a clear demarcation (Fig. 2d). In addition, the left and right vas deferens were also visualized. No obvious uterus, ovary or fallopian tube-like structure was visible. The laparoscopic examination further revealed that the patient’s right spermatic vessel was poorly developed and short (Fig. 2b, c). A diagnosis of undescended right testis and continuous SGF on the left side was made. The patent’s Internal Masculinization Score (IMS) was also assessed during the examination as part of our routine practice to summarize clinical features of his internal sex organ and to determine the degree of masculinization of the male infant, and he had an IMS score of 9 out of 10 [43].

As the European Association of Urology (EAU) guideline for paediatric urology recommends that “If a testis has not concluded its descent at the age of six months (corrected for gestational age), and since spontaneous testicular descent is unlikely to occur after that age, surgery should be performed within the subsequent year, and by age eighteen months at the latest” [47], we decided to perform the orchiopexy immediately. Considering the fact that the positions of the patient’s testes were high and in the abdominal cavity, and that the right spermatic vessel was poorly developed and short, it would be very difficult and risky to complete their descent into the scrotum through the internal rings and inguina or through the straight hernia triangle with single-stage Fowler-Stephen orchiopexy. It has been reported that although the risk of postoperative testicular atrophy was comparable between single-stage and 2-stage Fowler-Stephen orchiopexy, 2-stage Fowler-Stephen orchiopexy could lead to a more favorable testicular position in the scrotum [48]. As the patient’s mother had history of multiple miscarriages and received tocolytic treatment prior to the patient’s birth, the patient’s parents wanted the procedure to be as safe and effective as possible. Therefore, we decided to perform separate 2-stage laparoscopic staged Fowler-Stephen orchiopexies for both the left and the right sides. In order to reduce the risk of bilateral testicular atrophy, we decided against performing Stage 1 for both the left and the right sides simultaneously. Rather, we decided that Stage 1 for the right side should be performed first, if it was successful, Stage 2 for the right side and Stage 1 for the left side should then be performed months later, and if this was also successful, Stage 2 for the left side should be performed months after that. We completed stage 1 of laparoscopic staged Fowler-Stephen orchiopexy for the right testis without treating the SGF on the left side during the same single-site laparoscopic examination. Ultrasound monitoring immediately after the surgery revealed that the right testis had normal shape and good blood supply. B-mode ultrasound taken four months later (May 2, 2018) visualized a right testis of 1.2 × 0.6 × 0.6 cm with a normal shape and good blood supply and a testis-shaped hypoechoic mass of 1.5 cm × 0.8 cm extending to the spleen with good blood supply.

7 month later, on August 20, 2018, stage 2 of laparoscopic staged Fowler-Stephen orchiopexy for the right testis (descent of the right testis into the scrotum), separation of the left testis from the spleen as well as stage 1 of laparoscopic staged Fowler-Stephen orchiopexy for the left testis were performed. Ultrasound monitoring immediately after surgery revealed that both testes had normal shapes and good blood supply. B-mode ultrasound taken 4 months later (December 12, 2018) showed a right testis of 1.0 × 0.6 × 0.6 cm and a left testis of 1.1 × 0.5 × 0.4 cm, both with adequate blood supply.

**Fig. 3:**
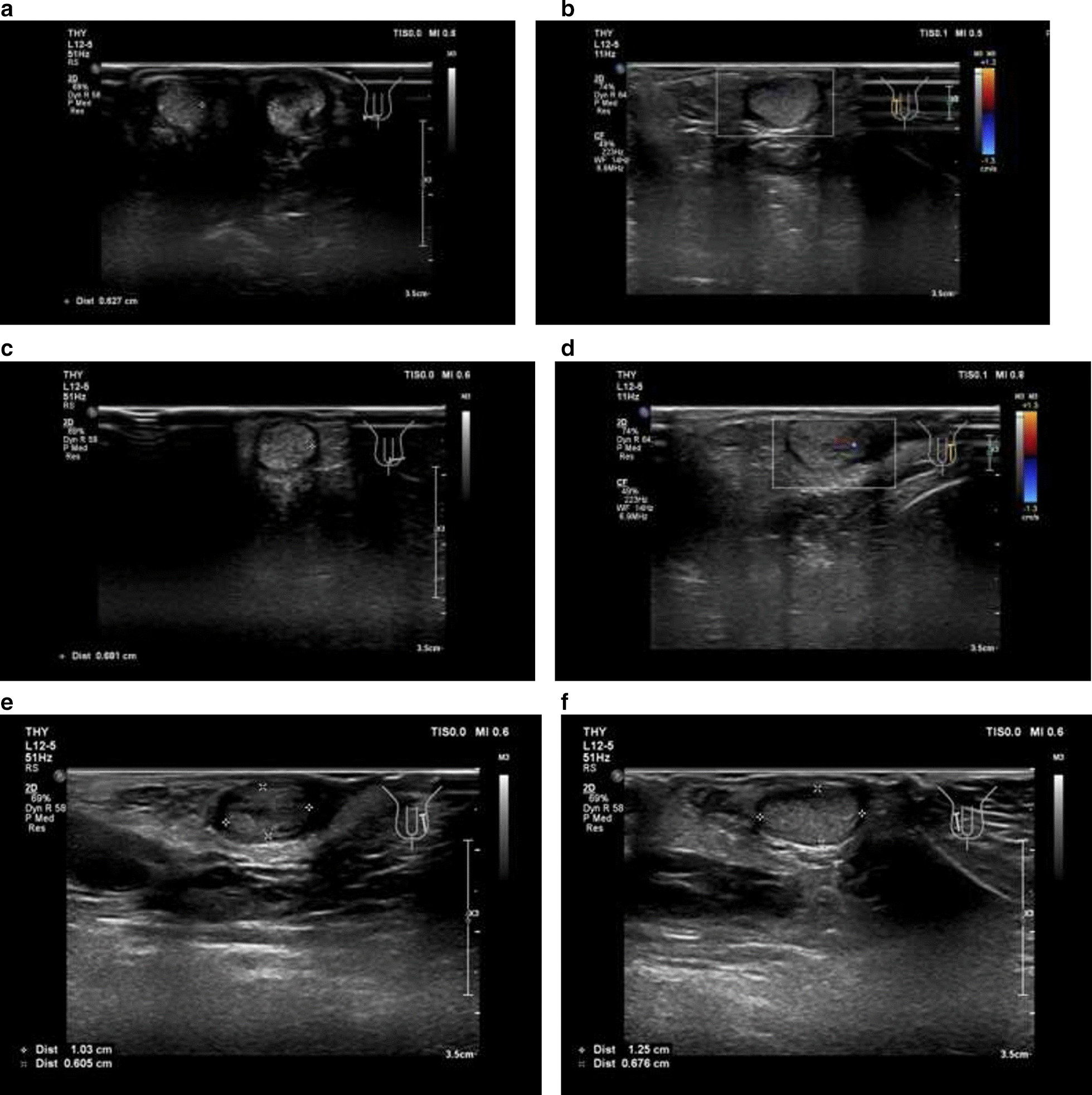
Post-operative ultrasound of the testes. **a** The right testis; **b** post-operative Doppler image of blood supply for the right testis; **c** left testis; **d** post-operative Doppler image of blood supply for the left testis; **e** and **f** follow-up ultrasound 1 year after the surgery showed that the left and right testes had a size of 1.0 × 0.6 × 0.6 cm and 1.3 × 0.7 × 0.7 cm, respectively, both with a homogeneous echogenic appearance and normal blood supply

Stage 2 of laparoscopic staged Fowler-Stephen orchiopexy for the left testis was performed 7 months later (March 12, 2019), the left testis descended into the scrotum. The residual spleen tissue was cut from the left testis (Fig. 2e) and post-operative biopsy confirmed that it was accessory spleen tissue. Ultrasound monitoring immediately after surgery revealed that both testis had normal shapes and good blood supply, it also visualized smooth tunica vaginalis and multiple hyperechoic spots without acoustic shadows indicating possible testicular microlithiasis (Fig. 3a–d). B-mode ultrasound taken 1 month later (April 20, 2019) revealed that both testes were in the scrotum and that the left and right testes had a size of 1.3 × 0.7 × 0.6 cm and 1.3 × 0.8 × 0.7 cm, respectively. Both testes had healthy blood supply and the spleen had a normal shape. The patient had an uneventful recovery and suffered no adverse or unanticipated events. Follow-up ultrasound 1 year after the surgery revealed no obvious abnormalities in the shapes of the testes or their blood supply (Fig. 3e, f) and the patient’s penis and scrotum had normal appearance (Fig. 1c).

## Discussion and conclusions

SGF is a very rare congenital anomaly, and only about 220 cases have been reported in literature [2, 5–41]. Carragher published a review of 123 cases in 1990 [3] and Malik et al*.* published a review of an additional 61 cases in 2013 [5]; We have examined 40 cases published in English after *Malik *et al*.* and summarized their data [2, 6–41] along with our present case (total 41 cases) in this study (Table 1). Detailed characteristics and treatments of each included case cannot be included in this article. Interested readers can find them in a Supplementary appendix online (Additional file 1: Table S1).

**Table 1:** Summary of characteristics and treatments of splenogonadal fusion (SGF) cases

	Number (N = 41)
*Age at diagnosis (years), n (%)*	
0–9	20 (48.78%)
10–19	6 (14.63%)
20–29	5 (12.20%)
30–39	6 (14.63%)
40–49	1 (2.44%)
50–59	2 (4.88%)
60–69	0 (0%)
70–79	1 (2.44%)
*Gender, n (%)*	
Male	40 (97.56%)
Phenotypical female (phenotypical sex reversal)	1 (2.44%)
*Side, n (%)*	
Left	41 (100%)
Right	0 (0%)
*Classification, n (%)*	
Continuous	19 (46.34%)
Discontinuous	22 (53.65%)
*Clinical presentation*	
Painful or painless inguinal / scrotal /testicular swelling/mass	28 (68.29%)
Cryptorchidism	11 (26.83%)^a^
Primary infertility	2 (4.88%) ^a^
A hypoplastic left heart and phenotypical sex reversal	1 (2.44%)
*Congenital anomalies associated with SGF, n (%)*	
Cryptorchidism	12 (29.27%)^b,c,d^
Inguinal hernia	5 (12.20%)
Limb and /or facial defects	2 (4.88%)^c^
Male infertility and/or azoospermia	2 (4.88%)^b^
Hypospadias	2 (4.88%)^d^
Left hip dysplasia	1 (2.44%)^c^
A hypoplastic left heart and phenotypical sex reversal	1 (2.44%)
None	21 (51.22%)
*Testis/ovary-sparing, n (%)*	
Yes	24 (58.54%)
No	14 (34.15%)
Unknown	3 (7.32%)

^a^One patient had both primary infertility and left sided cryptorchidism

^b^One patient had bilateral cryptorchidism, non-obstructive azoospermia and male infertility

^c^One patient had cryptorchidism, facial and limb deformities—short right femur, hip dysplasia and a syndromic face (Splenogonadal fusion-limb deformity syndrome)

^d^Both patients had cryptorchidism and hypospadias

SGF is a condition of male predominance (male/female ratio 14.3:1) [5], the reported male dominance is most likely due to underestimation of SGF occurrence in females as females’ gonads are inside the body and therefore less accessible for examination than the male gonads, in addition, there are fewer complications associated with female gonads than with male gonads [3, 5, 12]. Consistent with previous findings, 40 of the 41 cases (97.56%) in our review occurred in males, and the remaining 1 case occurred in a newborn who is phenotypical female (a 46, XY genotype despite a female phenotype) [8]. Also consistent with previous teaching that about 70% of SGF cases were pediatric cases [3, 5], 26 of the 41 cases (63.41%) in our study were reported in patients ≤ 19 years old (Table 1). The youngest patient was a newborn with a 46, XY genotype despite a female phenotype (phenotypical sex reversal) as well as a hypoplastic left heart, and this is the first reported SGF case associated with sex reversal [8].

SGF occurs between the 5^th^ and 8^th^ weeks of gestation. During the 5th and 6th weeks of gestation, the spleen develops from the splenic anlage in the left dorsal mesogastrium. At about the same time, the gonadal ridge is formed between the mesonephros and dorsal mesentery. As the embryonic gut rotates during week 5 of gestation, the dorsal mesogastrium rotates to the left, placing the splenic anlage into close proximity with the left urogenital fold which contains the gonadal mesoderm. Such close proximity remains until descent of the gonads and mesonephric involution during the 8^th^ week of gestation [1, 3, 5]. Several mechanisms have been proposed to explain the actual abnormal fusion between the splenic and gonadal tissues [5, 11]. The close proximity between the left gonad and spleen during embryogenesis explains the fact that SGF almost always occurs on the left side [1, 3, 5]. All of the 41 cases included in our study occurred on the left side as well (Table 1).

SGF can be classified into continuous and discontinuous SGF [3, 5]. Continuous SGF (55% of cases) is characterized by the presence of a cord of splenic or fibrous tissue connecting the spleen and gonad, and occasionally beads of splenic tissue could be found along the cord (splenic rosary bead), while in discontinuous SGF (45% of cases), there is no such connection. Rather, in discontinuous SGF, ectopic splenic tissue or accessory spleen is directly attached to the gonad without connecting to the native spleen [2, 3, 5, 7, 10]. During embryonic development, the descent of the testis could sometimes draw out the developing spleen fused to the testis into a long band (Continuous SGF) or to carry a portion of the splenic primordium down with the descending testis (Discontinuous SGF) [7]. 19 (46.34%) of the 41 cases in our study were continuous, and 23 (53.65%) cases were discontinuous (Table 1).

The most common malformations associated with SGF are cryptorchidism and inguinal hernia [1, 5, 12]. It has been reported that 31% of SGF patients had cryptorchidism or inguinal hernia [12], while Malik et al*.* found that 36% of SGF patients had cryptorchidism [5]. Other common anomalies include limb defects as well as craniofacial abnormalities such as micrognathia, most likely because active development of the limb bud and mandible occurs during 5^th^ and 8^th^ weeks of gestation, the same time as SGF [5]. Less common associations include cleft palate, Moebius syndrome, hypospadias, osteogenesis imperfecta, persistent mullerian duct syndrome, Potter syndrome, gastrointestinal malrotation, anal stenosis, and transverse testicular ectopia [5]. In our study, 20 of the 41 SGF cases (48.78%) were associated with other congenital anomalies, the most common being cryptorchidism (12 [29.27%]) and inguinal hernia (5 [12.20%]). Other less common associations included limb and facial defects, male infertility and/or azoospermia, hypospadias, left hip dysplasia, a hypoplastic left heart and phenotypical sex reversal (Table 1).

Our present case of left SGF in a male infant presented with bilateral cryptorchidism was not unusual for known SGF cases (Table 1) [3, 5, 12]. We used ultrasound to reach a preliminary diagnosis that was further confirmed by laparoscopy, this diagnostic process was also used in other reported SGF cases [12, 13, 20, 38]. What made our case unusual was that due to high positions of both testes (in the abdominal cavity), fusion of the left testis to the spleen and the poorly developed and short right spermatic vessel, it would be very difficult and risky to perform routine single stage orchiopexy on either side. Separate two-stage laparoscopic staged Fowler-Stephen orchiopexies for both sides were implemented. First, Stage 1 of the staged Fowler-Stephen orchiopexy for the right side was performed without treating the left side, and post-operative ultrasound visualized the right testis with a normal shape and good blood supply. Stage 2 for the right side (descent of right testis into the scrotum), separation of the left testis from the spleen as well as Stage 1 for the left side were performed months later, and post-operative ultrasound confirmed that both testes had normal shape and healthy blood supply. Finally, Stage 2 for the left side (descent of the left testis into the scrotum) was performed months after that. This approach reduced the risk of bilateral testicular atrophy associated with orchiopexy, allowed for a potentially more favorable testicular position in the scrotum [48], and spared the patient unnecessary orchiectomy.

Unlike previously reported SGF cases, we performed screening for genetic variant(s) related to disorder of sex development with panel based NGS test using MLPA. The NGS test revealed that the patient carried a missense mutation in SETBP1 (c.2608G>A [p.Gly870Ser]), a causative variation for Schinzel-Giedion syndrome (OMIM #269150). Schinzel-Giedion syndrome is a rare congenital disease characterized by serious intellectual disability, hypertrichosis, characteristic facial gestalt and various congenital malformations [44]. Patients with Schinzel-Giedion syndrome often die within several years after their birth [44]. The patient also carries a frameshift or nonsense mutation in SETBP1 known to cause autosomal dominant mental retardation-29 (OMIM #616078), a disease characterized by severe intellectual disability [45]. Finally, the patient has a mutation in OPHN1 known to cause X-linked mental retardation with cerebellar hypoplasia (OMIM #300486), a rare congenital anomaly characterized by “neonatal hypotonia with motor delay but no obvious ataxia, marked strabismus, early-onset complex partial seizures, and moderate to severe mental retardation” [46]. At the time of writing this article, the patient was a 3-year old who had had normal and healthy physical, cognitive, language and social-emotional development except for having weak eyesight in both eyes. Although visual impairment is one symptom of Schinzel-Giedion syndrome, the patient had not displayed any other symptoms of Schinzel-Giedion syndrome, and as such whether the weak vision displayed by the patient was due to Schinzel-Giedion syndrome is uncertain. Therefore the clinical significance of these mutations remains to be determined. We will follow-up with this patient regularly. Although it has been proposed that SGF is a genetic disorder [33], none of the previous reports on SGF performed panel-based NGS to identify genetic variations potentially related to various congenital disorders. Such testing could potentially allow for early detection and treatment of rare congenital anomalies.

SGF is often an incidental finding during exploration/surgery for scrotal swelling/mass, cryptorchidism or inguinal hernia [1, 5, 12]. In our study, the most common clinical presentations were painful or painless inguinal / scrotal /testicular swelling or mass (68.92%) and cryptorchidism (26.83%) (Table 1). SGF itself does not have characteristic features, its pre-operation diagnosis therefore could be difficult. In addition, lack of awareness of this rare condition could lead to its misdiagnosis [5, 12]. As a result, some patients with SGF underwent unnecessary orchiectomy [12]. In reality, there have been only 4 reported cases of SGF associated with testicular cancer, although cryptorchidism is associated with increased risk of testicular malignancy [6]. Up to 37% of SGF patients underwent unnecessary orchiectomy according to Carragher published in 1990 [3]. In our study, 14 of the 41 cases (34.15%) underwent orchiectomy [6, 8, 9, 12, 14–18, 22, 23, 35, 39], among them, 9 were potentially unnecessary [9, 12, 14–18, 39], reflecting a better awareness and more accurate diagnosis of SGF during the recent years. On the other hand, 21.95% of SGF patients still underwent unnecessary orchiectomy, indicating a continuing need for increasing awareness of this condition and better diagnostic protocols. Li et al*.* suggested a 3-step diagnostic protocol to rule out malignance and to diagnose and treat SGF when encountering an abnormal gonad [12]. First, SGF should be considered with a testicular mass existing from birth that has being growly slowly for years in a benign fashion [12]. Secondly, imaging techniques such as B-mode ultrasound, computed tomography (CT), MRI and Technicium-99m sulfur colloid liver-spleen scan could be employed to examine the mass for differential diagnosis. Among them, Technicium-99m sulfur colloid liver-spleen scan could detect accessory splenic tissue and thus could help to diagnose SGF when a surgeon has a high pre-operation suspicion of SGF [12]. Meanwhile, MRI is often first choice in detecting the position and shape of the testes and ruling out other congenital anomalies. It is reliable and accurate in detecting/ruling out a testicular or scrotal lesion, localizing the lesion, and differentiating intratesticular and extratesticular lesions [49]. In addition, by using different sequences and administering gadolimium, the pattern of scrotal disorder can be characterized and testicular lesions could be classified [49]. At the same time, it could also detect or rule out anomalies in a patient’s other key organs such as liver, spleen, kidneys and brain. However, MRI is expensive and could be hard to perform on infants who need to be sedated. In our case, MRI failed to detect our patient’s testes, possibly because too much coats on the infant while being imaged, poor skills of the technician or insufficient bowel preparation. Meanwhile, ultrasound is inexpensive and high-resolution ultrasound employing gray-scale and color-encoded techniques is considered by some to be an accepted standard for scrotal imaging [49]. In our practice, ultrasound is used for the purpose of preliminary screening for congenital anomalies. It could help to diagnose continuous SGF when a cord connecting the spleen to the testicle is visualized. On the ultrasound, the splenic tissue is usually visualized as a well-encapsulated, extra-testicular homogenous hypoechoic or isoechoic mass [5, 12]. In addition, Doppler ultrasound could monitor blood flow to the testes [50]. In our case, ultrasound visualized both testes in the lower abdominal cavity as well as the upper margin of the left testicle as a hypoechoic mass extending to the spleen indicating an undescended right testis and a possible SGF on the left side. However, it has been suggested that in cases where ultrasound produced inconclusive findings, MRI could be valuable [49]. Li et al*.* further suggested that the third step was, when unsure, an intra-operation biopsy should be performed to determine the nature of the mass [12]. In any case, diagnostic laparoscopy is recommended as it is safe, reliable and very accurate in diagnosing and treating an impalpable testis [5]. When a definite diagnosis of SGF is made, it is sufficient to completely excise the splenic tissue and preserve the testes especially in young patients [5]. For some, surgery may not even be necessary [12]. In some instances as demonstrated by our case, when routine single stage orchiopexy was not feasible or risky on either the left or the right side, separate two-stage laparoscopic staged Fowler-Stephen orchiopexies on the left and right sides could be performed to prevent unnecessary orchiectomy. Our case report in this sense provides a scenario in which unnecessary orchiectomy could be avoided although whether such treatment strategy could be widely applied could only be determined by future cases.

In conclusion, SGF is a rare congenital anomaly often diagnosed incidentally during exploration/surgery for scrotal swelling/mass, cryptorchidism or inguinal hernia in young patients. Surgeons, especially pediatric surgeons should be aware of this condition to avoid unnecessary, life-altering radical orchiectomy. In some cases, when routine single stage orchiopexy is not feasible or risky for either side, separate two-stage laparoscopic staged Fowler-Stephen orchiopexies could be performed on both the left and right sides to avoid unnecessary orchiectomy.

## Supplementary information

**Additional file 1:** Table S1. Characteristics and treatments of included splenogonadal fusion (SGF) case.

## Data Availability

The datasets used and/or analyzed during the current study are available from the corresponding author on reasonable request.

## Abbreviations

SGF: splenogonadal fusion
MRI: magnetic resonance imaging
EMS: External Masculinization Score
NGS: next generation sequencing
MLPA: multiple ligation-dependent probe amplification
SETBP1: SET binding protein 1
OPHN1: oligophrenin-1
IMS: Internal Masculinization Score
CT: computed tomography
