# Supplementary material for: Splenogonadal fusion: a case report and review of the literature
Source: BMC Urol. 2021 Feb 3;21:16. doi: 10.1186/s12894-021-00781-z (PMC7860507; doi:10.1186/s12894-021-00781-z)
Supplement: Supplementary file 1 — Additional file 1: Table S1. Characteristics and treatments of included splenogonadal fusion (SGF) case. [file 12894_2021_781_MOESM1_ESM.docx]

**Additional file 1: Table S1** Characteristics and treatments of included splenogonadal fusion (SGF) cases

| References | Age at diagnosis | Gender | Classification | Left or right side | Clinical presentation | Congenital anomalies associated with SGF | Treatment |
| --- | --- | --- | --- | --- | --- | --- | --- |
| Lopes *et al.* 2012 [6) | 36 years | Male | Discontinuous | Left | 8 years of primary  infertility | Bilateral cryptorchidism, non-obstructive azoospermia and male infertility. | A left orchiectomy† combined with immediate bench testicular microdissection; a right orchidopexy along with biopsies of the upper, mid and lower pole of the testis was scheduled. In the surgery, the testis could be brought only into the external inguinal ring, where it was fixed in place |
| Patre *et al.* 2012 [40) | 18 years | Male | Discontinuous | Left | A large left scrotal swelling since birth which was progressively increasing in size | Limb defect - Syndactyly detected over the right middle  and ring finger with right lower limb shortening | Testis-sparing surgery |
| Speare *et al.* 2012 [8) | Newborn | Phenotypical female (phenotypical sex reversal) | Continuous | Left | A hypoplastic left heart, a 46,XY genotype despite a female phenotype (phenotypical sex reversal) | A hypoplastic left heart, a 46,XY genotype despite a female phenotype (phenotypical sex reversal) | Gonadectomy‡ |
| Duhli *et al.* 2013 [9) | 28 years | Male | Discontinuous | Left | Left testicular pain and progressive swelling for 3 months | None | A left high inguinal orchiectomy and right orchidopexy |
| Ferrón *et al.* 2013 [10) | 2 years | Male | Discontinuous | Left | 2-month history of painless left scrotal mass with no associated skin discoloration but a slight increase in scrotal size | None | At surgery there was a left paratesticular mass that was easily separated from the left testicle, allowing complete resection |
| Jayasundara *et al.* 2013 [25) | 5 months | Male | Continuous | Left | A scrotal mass noted  during an episode of viral fever | None | The SGF was divided using bipolar  electrocautery, and the cord of the splenic tissue was divided deep to the deep inguinal ring after ligation. The resected segment was sent for histological assessment. Herniotomy was done and the testicle was repositioned into the scrotum |
| Li *et al.* 2013 [12) | 6 years | Male | Discontinuous | Left | A painless palpable mass in the left scrotum. | None | Testis sparing surgery |
|  | 7 years | Male | Continuous | Left | A left palpable inguinal mass | Left inguinal congenital hernia | Laparoscopy - The proximal cord-like tissue was ligatured prior to being resected |
|  | 2 years | Male | Discontinuous | Left | Bilateral undescendent testes - Bilateral cryptorchidism | Bilateral cryptorchidism and hypospadias | Laparoscopy - The removal of the left testis-like mass was followed by the high ligature of the spermatic vein. The right testicle was pulled down and fixed in a secondary surgery. |
|  | 12 years | Male | Discontinuous | Left | Bilateral undescendent testes – Bilateral cryptorchidism | Bilateral cryptorchidism and hypospadias | The right testicle was pulled down and fixed, while the left, testis-like mass was removed |
| Liu *et al.* 2013 [13) | 6 years | Male | Continuous | Left | Painless left scrotal mass. | None | The sausage shaped  structure uncovered by  diagnostic laparoscopy was dissected from the testis and spleen without injury. A laparoscopic pediatric processus vaginalis suturing ligation was then performed. Histopathology  reported the specimen to be of normal splenic tissue. |
| Zhang *et al.* 2013 [22) | 31 years | Male | Continuous | Left | A two-year history of a painless left testicular mass that was progressively increasing in size for one year | None | Considering that the right testis was normal, the whole mass with the affected testicle in the left scrotum was surgically removed§ |
| Bal *et al.* 2014 [15) | 20 years | Male | Discontinuous | Left | Left scrotal pain and a palpable mass. The pain started after a vigorous physical exercise session, and the palpable mass had been present since childhood. | None | Left inguinal orchiectomy due to suspected malignant or benign testicular tumor |
| Bosnalı *et al.* 2014 [26) | 7 years | Male | Continuous | Left | Bulging in the left groin that had existed  for 3 years – diagnosis of left indirect inguinal hernia | Left indirect inguinal hernia | SGF found incidentally during indirect inguinal hernia repair, the testicle was preserved during excision. Laparoscopic exploration was helpful in identifying the isolated polysplenia as the origin of continuous-type SGF, and in excising the cord-like attachment proximally |
| Chiaramonte *et al.* 2014 [33) | 12 years | Male | Discontinuous | Left | Left small scrotal mass | None | After frozen section mass was excised sparing testis |
| Kocher *et al.* 2014 [18) | 35 years | Male | Discontinuous | Left | A painless left scrotal mass | None | A left inguinal orchiectomy |
| Kumar *et al.* 2014 [19) | 25 years | Male | Continuous | Left | A complaint of primary infertility and his right scrotal testis was atrophied and left intra-abdominal undescended  testis | Cryptorchidism and infertility | Laparoscopic assessment suggestive SGF, excision and left orchidectomy were performed |
| Lakshmanan *et al.* 2014 [20) | 6 years | Male | Continuous | Left | Left inguinoscrotal swelling | Left  congenital inguinal hernia | Explorative laparotomy and a diagnosis of SGF was made. The connecting band was sectioned and orchidopexy of the left testicle was performed. |
| Sountoulides *et al.* 2014 [21) | 31 years | Male | Continuous | Left | A palpable, solid, hard, but painless nodule on the upper pole of his left testicle | None | The smaller lesion and the cord-like structure were dissected, while the larger lesion attached to the testis was dissected off the testicular parenchyma using cautery. The surface of the testicle was then reconstructed and the testicle was repositioned into the scrotal sac |
| Croxford *et al.* 2015 [14) | 18 years | Male | Discontinuous | Left | A small hard  nodule at the upper pole of his left testis diagnosed as a testicular tumor | None | A left radical inguinal orchidectomy with  insertion of a testicular prosthesis |
| Shadpour *et al.* 2015 [23) | 17 years | Male | Continuous | Left | Bilateral impalpable testes - Bilateral cryptorchidism | Bilateral cryptorchidism. | Laparoscopic exploration – Right gonad was successfully pexed into the right hemiscrotum by  combining one-stage Fowler-Stephens and Prentiss maneuvers. On the left side, the rosary was freed and excised although salvaging the testis was not at all an option as explained above¶ |
| Trottmann *et al.* 2015 [24) | 31 years | Male | Continuous | Left | Severe pain and swelling of the left scrotum | None | Scrotal exploration, suspicious part of left testis was removed for intraoperative frozen section and the rest of the testis was retained |
| Celik *et al.* 2016 [11) | 14 months | Male | Continuous | Left | Left undescended testis –left sided cryptorchidism | Left cryptorchidism, facial and limb deformities – short right femur, hip dysplasia and a syndromic face (Splenogonadal fusion-limb deformity syndrome) | SGF found during a routine orchiopexy, and confirmed by a laparoscopic examination. The splenic tissue was dissected, a gross arterial connection restricting the descent was identified and ligated, and orchiopexy was carried out |
| Harris *et al.* 2016 [16) | 55 years | Male | Discontinuous | Left | Left sided upper pole testicular mass. | None | Left sided radical inguinal orchiectomy |
| Jakkani *et al.* 2016 [27) | 16 years | Male | Discontinuous | Left | Bilateral cryptorchidism | Bilateral cryptorchidism | A large pinkish mass with vascular pedicle in the left lumbar and iliac region which was resected. Right inguinal testis was managed by laparoscopic Stephen-Fowler stage procedure by high ligation of testicular artery |
| Uglialoro *et al.* 2016 [28) | 45 years | Male | Discontinuous | Left | 10-year history of an indolent, enlarging, painless left testicular mass | None | An inguinal approach to a scrotal exploration and partial orchiectomy. |
| Akama *et al.* 2017 [29) | 76 years | Male | Continuous | Left | A 10-year history of left inguinal swelling and a one-month history of occasional pain – Diagnosis of left direct inguinal hernia | None | A laparoscopic transabdominal preperitoneal hernia repair operation in which the cord was cut for mesh replacement and an accurate diagnosis of the mass as SGF was made |
| Abokrecha *et al.* 2017 [2) | 18 months | Male | Discontinuous | Left | Bilateral empty scrotum since birth - Bilateral impalpable undescended testes (cryptorchidism) | Bilateral cryptorchidism | Left side scrotal orchidopexy after an incisional biopsy of the mass confirmed SGF. As the patient had only one testicle with a splenic tissue remnant in the upper pole, the plan for meticulous dissection to excise the residual splenic tissue was made. |
| Huang *et al.* 2017 [30) | 4 years | Male | Continuous | Left | Bilateral undescended testes – diagnosed as bilateral cryptorchidism | Bilateral cryptorchidism | SGF diagnosed during laparoscopic exploration, the spleen cord was resected and Fowler‑Stephens orchiopexy was implemented at the same time. |
| Li *et al.* 2017 [31) | 2 years | Male | Continuous | Left | Recent painless recent swelling of the left scrotum | None | Intraoperative frozen section confirmed SGF, subsequently, the far end of cordlike tissue was ligated near the internal inguinal ring, and the ectopic spleen was separated from the testis and returned to the abdomen. The left testicle was preserved and lowered to the scrotum |
| Preece *et al.* 2017 [32) | 18 months | Male | Continuous | Left | Right cryptorchidism  and left retractile testicle. | Right cryptorchidism  and left retractile testicle | Open excision of the adherent splenic tissue, orchiopexy of the testicle via an open inguinal approach, and the discontinuous splenules were left in situ |
| Karray *et al.* 2018 [17) | 38 years | Male | Discontinuous | Left | A painless palpable left scrotal mass | None | A radical inguinal orchiectomy |
| Srinivasa Rao *et al.* 2018 [34) | 6 years | Male | Continuous | Left | An acutely painful left inguinal swelling mimicking a strangulated inguinal hernia | Indirect left inguinal hernia | An inguinal procedure - The firm swelling was excised by dividing the fibrous cords from the upper pole of the testis distally and as high as possible proximally. Herniotomy was done. |
| Shakeri *et al.* 2018 [35) | 4 years | Male | Discontinuous | Left | Nonprogressive  unilateral scrotal enlargement of a few months duration | Left inguinal hernia and inguinal canal accessory spleen | Open surgery with a presumptive diagnosis of testicular neoplasm. Radical orchiectomy was performed because there  was hardly any testicular parenchyma to be salvaged; and then, surgery was finished by performing a herniotomy# |
| Zhou *et al.* 2018 [36) | 9 years | Male | Discontinuous | Left | A left-sided scrotal mass. | None | The frozen section revealed splenic tissue with no features of malignancy seen. The remainder of the  mass was excised and the testis was returned to the scrotum |
| Chen *et al.* 2019 [37) | 12 month | Male | Discontinuous | Left | Left cryptorchidism | Left cryptorchidism | SGF found during a left orchiopexy, the splenic tissue was carefully dissected from the testicle and cord structure and released into retroperitoneum |
| Grosu *et al.* 2019 [38) | 53 years | Male | Discontinuous | Left | Unclear swelling of the left testicle | None | Surgical scrotal exploration wherein the lesion was removed and the rest of the testis preserved. |
| Mann *et al.* 2019 [39) | 22 years | Male | Discontinuous | Left | A hard mass within the upper pole of his left testicle with associated  lower abdominal pain which radiated into his left testicle. Examination revealed two discrete palpable masses, one near the epididymis and the other unable to be differentiated from the testicle itself | None | A radical orchidectomy |
| Xiang *et al.* 2019 [7) | 5 months | Male | Continuous | Left | A history of obvious left scrotal swelling for 1 month, which progressively worsened 10 h before the hospital visit – a soft and nonreturnable mass in the left scrotum. | Inguinal hernia | Laparoscopic exploration, the left scrotum mass was removed completely. The intact left testis was preserved, while the epididymis was retained as much as possible so as to not affect the function of the epididymis. |
| Seager *et al.* 2020 [41) | 25 years | Male | Discontinuous | Left | Balanitis and a scrotal swelling separate to the left testicle due to a paratesticular mass | None | Surgically explore the lesion with a view to biopsy or removal, the lesion, along with the testicle, was left in situ. Intra-lesional biopsies were obtained and closure performed. |
| Present case | 8 months | Male | Continuous | Left | Bilateral cryptorchidism | Bilateral cryptorchidism | Right gonad was successfully pexed into the right hemiscrotum by laparoscopic staged Fowler-Stephens orchipexy; on the left side, the spleen was separated from the testis which was pexed into the left hemiscrotum by laparoscopic staged Fowler-Stephens orchipexy. |

†A left orchiectomy was necessary because “this patient had a left atrophic testis with probable embryological attachment alterations associated with azoospermia, due to testicular dysgenesis, and a statistically higher risk of testicular malignancy” [6].

‡ A gonadectomy was necessary because the patient has a 46,XY genotype despite a female phenotype [8].

§An orchiectomy was necessary ”when contralateral testicle is normal and the affected testicular appeared significantly atrophy” [22].

¶A left orchiectomy was necessary because “On the left side, however, a smaller 20mm long gonad resembling the testis was encountered distally in the pelvis but with anomalous total disjunction from the hypoplastic vasoepididymal structures. Because there was no continuity between the left gonad and epididymis, the only blood supply to the organ was via internal spermatic vessels, making this short spermatic vascular pedicle indispensable, and Fowler orchiopexy is not an option, even if staged”, and “In our 17-year-old case, salvaging the testis was not at all an option as explained above.” [23].

#Radical orchiectomy was performed because there was hardly any testicular parenchyma to be salvaged” because “the testicular remnant was very much atrophied and later found to be of primitive histology.” [35].
